# miR-590-3p and Its Downstream Target Genes in HCC Cell Lines

**DOI:** 10.1155/2019/3234812

**Published:** 2019-11-03

**Authors:** Mennatallah Elfar, Asma Amleh

**Affiliations:** ^1^Biotechnology Program, The American University in Cairo, Cairo, Egypt; ^2^Biology Department, The American University in Cairo, Cairo, Egypt

## Abstract

miRNAs are small non-coding RNA sequences of 18-25 nucleotides. They can regulate different cellular pathways by acting on tumor suppressors, oncogenes, or both. miRNAs are mostly tissue-specific, and their expression varies depending on the cancer or the tissue in which they are found. hsa-miR-590-3p was found to be involved in several types of cancers. In this study, we identified potential downstream target genes of hsa-miR-590-3p computationally. Several bioinformatics tools and more than one approach were used to identify potential downstream target genes of hsa-miR-590-3p. CX3CL1, SOX2, N-cadherin, E-cadherin, and FOXA2 were utilized as potential downstream target genes of hsa-miR-590-3p. SNU449 and HepG2, hepatocellular carcinoma cell lines, were used to carry out various molecular techniques to further validate our *in silico* results. mRNA and protein expression levels of these genes were detected using RT-PCR and western blotting, respectively. Co-localization of hsa-miR-590-3p and its candidate downstream target gene, SOX2, was carried out using a miRNA in situ hybridization combined with immunohistochemistry staining through anti-SOX2. The results show that there is an inverse correlation between hsa-miR-590-3p expression and SOX2 protein expression in SNU449. Subsequently, we suggest that SOX2 can be a direct downstream target of has-miR-590-3p indicating that it may have a role in the self-renewal and self-maintenance of cancer cells. We also suggest that CX3CL1, E-cadherin, N-cadherin, and FOXA2 show a lot of potential as downstream target genes of hsa-miR-590-3p signifying its role in epithelial-mesenchymal transition. Studying the expression of hsa-miR-590-3p downstream targets can enrich our understanding of the cancer pathogenesis and how it can be used as a therapeutic tool.

## 1. Introduction

Cancer is considered a worldwide epidemic, as it is the second leading cause of death worldwide. According to the World Health Organization, cancer is responsible for around 9.6 million deaths in 2018 only [1]. Liver cancer is the second leading cause of cancer deaths worldwide [2]. According to the American Cancer Society, since 1980, liver cancer incidence has more than tripled. Every year, approximately 700,000 new cases of liver cancer are diagnosed. According to GLOBOCAN, the less developed countries account for 83% of these cases. Liver cancer is male predominant [3].

Hepatocellular carcinoma (HCC), also known as malignant hepatoma, is the most common primary tumor of liver cancer cases [4]. It usually occurs secondary to liver cirrhosis due to viral hepatitis infection (HBV and HCV), alcoholism, or exposure to high levels of aflatoxin-b1 (AFB). HCC is heterogeneous and a number of factors contribute to the disease progression, starting from tumor initiation to metastasis [5]. These factors include the tumor's microenvironment, hypoxia, inflammation, and oxidative stress [6]. Cytokines and reactive oxygen species released in the organ's microenvironment due to chronic inflammation and oxidative stresses, respectively, result in gradual accumulation of mutations that change the hepatocytes genetically, altering gene expression and affecting various signaling pathways causing liver damage and eventually cancer development [7].

Small non-coding RNA sequences, known as microRNAs (miRNAs), are 18-25 nucleotides. miRNAs can posttranscriptionally regulate different cellular pathways including cellular differentiation, growth, proliferation, metabolism, angiogenesis, regeneration, survival, apoptosis, and tumorigenesis [8].

Most miRNA genes are located on intergenic regions (non-coding regions) of the nuclear DNA and are expressed in the cytoplasm through the following process. In the nucleus, RNA polymerase II transcribes a long primary miRNA (pri-miRNA) with a hairpin-like structure. Drosha then converts it to precursor miRNA (pre-miRNA), a 70-nucleotide stem loop, followed by its transport to the cytoplasm through the action of Exportin 5, where it meets Dicer and is cleaved into 22-nucleotide double-stranded miRNAs. Then, it is separated into two single-stranded miRNAs. The sense (passenger) strand is degraded while the antisense (guide) strand binds to RNA-induced silencing complex (RISC). The interaction between the 5′ seed sequence of the miRNA and the 3′-untranslated region of the mRNA determines the effect of the miRNA, whether degradation of the mRNA or the inhibition of its translation [9].

According to miRBase, there are 1917 precursor and 2654 mature human miRNAs [10]. Various miRNAs can act as tumor suppressors, oncogenes, or both through the regulation of their target genes. They are tissue-specific, meaning they can be upregulated or downregulated, depending on the cancer or the tissue in which they are found [11]. hsa-miR-590-3p is a good example of one miRNA that is upregulated in a specific cancer and downregulated in another.

In this study, we focused on hsa-miR-590-3p's downstream target genes. We used various bioinformatics analyses to identify the potential downstream target genes of hsa-miR-590-3p and to predict their function in relation to HCC. Then, we employed various molecular techniques to access the levels of the potential downstream target genes. The main aim of the study is to assess the levels of hsa-miR-590-3p and the role of its downstream target genes in HCC.

## 2. Material and Methods

### 2.1. *In Silico* Analysis

Several bioinformatics tools were used to predict the potential downstream target genes of hsa-miR-590-3p. For the primary screening, five databases were used (TargetScan [12] (http://www.targetscan.org), miRanda-mirSVR [13] (http://www.microrna.org), miRDB [14] (http://mirdb.org/), miRTarBase [15] (http://mirtarbase.mbc.nctu.edu.tw/php/index.php), and Diana Tools [16] (http://diana.imis.athena-innovation.gr)) to achieve a dataset of potential downstream target genes of hsa-miR-590-3p from each database. For the function prediction, we used the Functional Assignment of MicroRNAs via Enrichment (FAME) software [17] (http://acgt.cs.tau.ac.il/fame/index.html) to predict the potential functions of hsa-miR-590-3p through the prediction of the potential functions of its potential downstream target genes. Alignment of the mRNA of potential downstream target genes against hsa-miR-590-3p was carried out using miRanda-mirSVR [13] (http://www.microrna.org). The mRNA and protein expression of these genes were assessed computationally using The Human Protein Atlas [18] (http://www.proteinatlas.org/) and Expression Atlas [19] (http://www.ebi.ac.uk/gxa), respectively.

### 2.2. Cell Lines and Cell Culture

The human hepatocellular carcinoma cell lines, HepG2 and SNU449, were used. HepG2 is an early-stage liver cancer cell line while SNU449 is an HBV-infected intermediate stage liver cancer cell line. Both cell lines were a kind gift from Dr. Mehmet Ozuturk at the Department of Molecular Biology and Genetics, Bilkent University, Turkey. Cells were cultured in RPMI 1640 (Lonza, USA) media supplemented with 10% FBS (Gibco, USA) and 5% penicillin-streptomycin antibiotic (Lonza, USA). They were maintained at 37°C and 5% CO_2_ in a humidified atmosphere. Cells were passaged and used for experiments during their logarithmic growth phase.

### 2.3. Semiquantitative Reverse Transcription-Polymerase Chain Reaction (RT-PCR)

Total RNA was extracted from HepG2 and SNU449 cells using TRizol Reagent (Invitrogen, USA) following the manufacturer's protocol. 0.5 *μ*g RNA was used to synthesize cDNA using the RevertAid First Strand cDNA Synthesis Kit (Thermo Scientific, USA) according to the manufacturer's protocol. mRNA expression was determined using semiquantitative RT-PCR. MyTaq Red DNA Polymerase (Bioline, UK) was used to perform the PCR reactions using GAPDH as an endogenous control. Specific primers (Invitrogen, USA) were designed using Primer3 [20] (http://primer3.ut.ee) for each gene. PCR conditions used were the same among the genes except for the annealing temperatures and the number of cycles. PCR conditions are as follows: Step 1: initiation at 94°C for 3 minutes. Step 2: denaturation at 94°C for 30 seconds, annealing at specific temperatures for each primer for 30 seconds, and extension at 72°C for 45 seconds; Step 2 is repeated for a specific number of cycles for each primer. Step 3: final extension at 72°C for 10 minutes. Primer sequences, annealing temperatures, number of cycles, and PCR amplicon sizes are listed in [Supplementary-material supplementary-material-1].

### 2.4. Western Blotting Analysis

Total protein was extracted from HepG2 and SNU449 cells using 1x Laemmli lysis buffer (50 mM Tris/HCl, pH 6.8, 2% SDS, and 10% glycerol) supplemented with 1x Halt Protease Inhibitor Cocktail (Thermo Scientific, USA). The Pierce BCA Protein Assay Kit (Thermo Scientific, USA) was used to quantify the total extracted protein according to the manufacturer's protocol. 30 *μ*g protein was used to perform western blotting. Protein was separated on a 10% SDS-polyacrylamide gel and electrotransferred onto a nitrocellulose membrane (Thermo Scientific, USA). The membrane was blocked using 5% non-fat dry milk in 1x TBST (0.01% Tween 20 in 1x TBS buffer) and then incubated with specific primary antibodies followed by the secondary antibodies. The BCIP/NBT phosphatase colorimetric substrate (KPL, USA) was used for detection. GAPDH and *β*-tubulin were used as endogenous controls.

Antibodies were diluted in 5% non-fat dry milk in 1x TBST. Primary antibodies used were as follows: anti-GAPDH (1 : 10,000, ab8245, Abcam, UK), anti-*β*-tubulin (1 : 20,000, T7816, Sigma, USA), anti-SOX2 (1 : 2000, PA1-16968, Thermo Scientific, USA), and anti-Vimentin (1 : 1000, ab8978, Abcam, UK). Secondary antibodies used were as follows: ReverseAP Phosphatase labeled Goat anti-Mouse IgG (H+L) Conjugate (1 : 20,000, 4751-1806, KPL, USA) and ReverseAP Phosphatase labeled Goat anti-Rabbit IgG (H+L) Conjugate (1 : 10,000, 4751-1516, KPL, USA).

### 2.5. *In Situ* Hybridization-Immunocytochemistry (ISH-ICC)

For the codetection of hsa-miR-590-3p and one of its downstream target genes (SOX2), ISH-ICC was carried out on SNU449 cells. Cells were seeded into 24-well cell culture plates and incubated till 70% confluency was reached. Fixation of the cells was carried out using 100% methanol. For the ISH, the cells were incubated with DIG-labeled probes for miR-590-3p (hsa-miR-590-3p miRCURY LNA Detection probe, Exiqon, Denmark), U6 (as a positive control probe), and scrambled probes (as a negative control) (miRCURY LNA microRNA Detection ISH Buffer and Controls kits, Exiqon, Denmark) for 1 hour at 54°C. Blocking was done using a blocking solution rich in 7.5% BSA Fraction V (Gibco, USA), and then, the cells were incubated with anti-DIG phosphatase labeled antibody (1 : 800, 11 093 274 910, Roche, Germany). The BCIP/NBT phosphatase colorimetric substrate (KPL, USA) was used for hsa-miR-590-3p detection. For ICC, blocking was performed again and the cells were incubated with anti-SOX2 (1 : 250, PA1-16968, Thermo Scientific, USA), followed by the secondary antibody, Goat anti-rabbit IgG (H+L) DyLight 488 Conjugated (1 : 250, 35552, Thermo Scientific, USA). The nuclei were stained with DAPI (1 : 1000 in PBS, KPL, USA).

### 2.6. Statistical Analysis

PCR and western blotting analysis results were quantified and normalized against an endogenous control using ImageJ Software [21] (https://imagej.nih.gov/ij/). Data is presented as mean ± standard deviation (SD) from three independent experiments. All statistical comparisons were done using Prism GraphPad 7.0 [22] (http://www.graphpad.com/). For the analysis of the difference between multiple experimental groups with a single variable, one-way ANOVA (with a Bonferroni posttest) was used. *P* values less than 0.05 are considered significant (^∗^*P* value < 0.05, ^∗∗^*P* value < 0.01, and ^∗∗∗^*P* value < 0.001).

## 3. Results

### 3.1. Prediction of Potential Downstream Target Genes of hsa-miR-590-3p

Prediction of the potential downstream target genes of hsa-miR-590-3p was carried out using five databases (TargetScan, miRanda-mirSVR, miRDB, miRTarBase, and Diana Tools). Each database uses a different scoring system based on different molecular and bioinformatics techniques. TargetScan uses the conserved sites of the target genes that match miRNA's seed region to predict the miRNA's target genes [23]. Through TargetScan, 8611 potential target genes of hsa-miR-590-3p were obtained ([Supplementary-material supplementary-material-1]). miRanda-mirSVR uses a two-step strategy to predict potential target genes of miRNAs. Primarily, the program aligns the miRNA sequence against mRNA sequences and produces a score based on the complementarity through A : U and G : C matches, followed by the usage of high-scoring alignments, meaning they passed a certain threshold, to calculate their thermodynamic stability [13]. Through miRanda-mirSVR, 21,123 potential downstream target genes of hsa-miR-590-3p were obtained. miRDB uses MirTarget, a bioinformatics tool, to predict potential target genes. MirTarget was developed by the analysis of thousands of miRNA-target interactions obtained from high-throughput sequencing experiments [24]. Through miRDB, 1590 potential target genes of hsa-miR-590-3p were obtained ([Supplementary-material supplementary-material-1]). miRTarBase was established on the collection of miRNA-target interactions (MTIs) from the previous literature and validating them experimentally using next-generation sequencing (NGS), microarray, western blotting, and reporter assay [15]. Through miRTarBase, 447 potential target genes of hsa-miR-590-3p were obtained ([Supplementary-material supplementary-material-1]). Diana TarBase was established using specific and high-throughput experiments to predict miRNA-gene interactions [16]. Through Diana TarBase, 4576 potential target genes of hsa-miR-590-3p were obtained ([Supplementary-material supplementary-material-1]). All potential downstream target genes of hsa-miR-590-3p from all 5 databases were placed in a pivot table, and a list of 362 common genes (primary screening) was obtained ([Supplementary-material supplementary-material-1]).

To get further insight on hsa-miR-590-3p's function, we performed the functional prediction analysis of its downstream target genes using FAME software. FAME analysis is founded on a collection of miRNA pathways and miRNA process association that has been verified experimentally [17]. FAME identified many functions and the downstream target genes of hsa-miR-590-3p that perform these functions. We chose cancer pathogenesis-related functions to narrow down our study ([Supplementary-material supplementary-material-1]). These functions include response to DNA damage stimulus, DNA repair, cell-cell adhesion, nucleotide excision repair, and DNA damage response and signal transduction. Using this approach (function prediction), thirty-four potential target genes of hsa-miR-590-3p were achieved.

**Figure 1:**
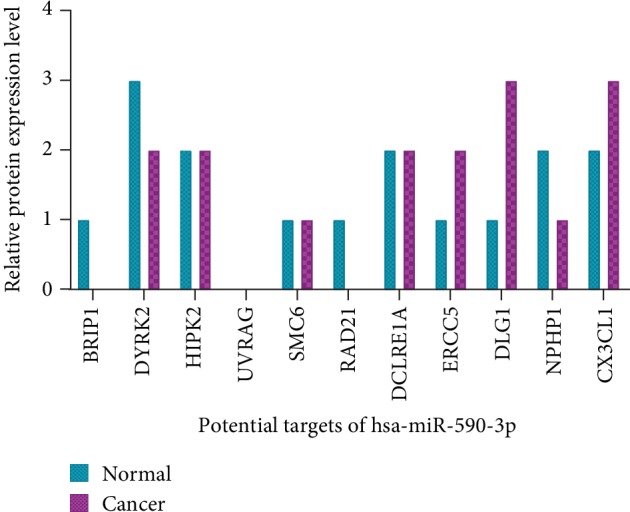
Protein expression of potential targets of hsa-miR-590-3p in normal versus cancerous liver tissue using The Human Protein Atlas. Diagram of the protein expression of the potential downstream target genes chosen for further analysis in normal liver tissue versus cancerous liver tissue. Scale: 0: not detected; 1: low; 2: medium; and 3: high.

**Figure 2:**
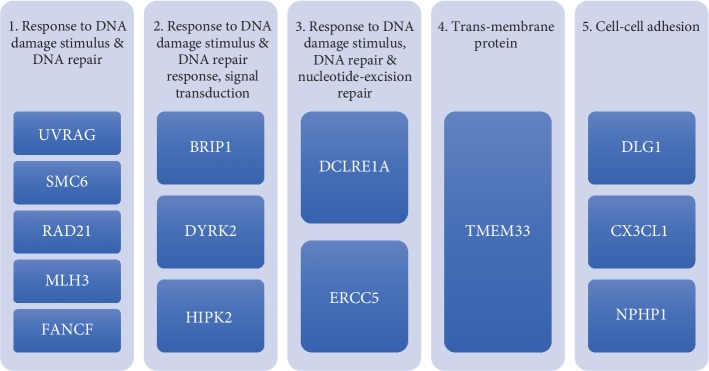
Classification of the fourteen genes. The fourteen genes chosen for further analysis categorized based on the functions they share according to the FAME software.

Thirty-two genes of the thirty-four genes from the function prediction approach were found in the list from the primary screening approach. From these thirty-two genes, fourteen genes were chosen based on the results obtained from The Human Protein Atlas [18] (Figure 1) and previous literature. These genes are BRIP1, CX3CL1, DCLRE1A, DLG1, DYRK2, ERCC5, FANCF, HIPK2, MLH3, NPHP1, RAD21, SMC6, TMEM33, and UVRAG. The genes were categorized according to the functions they share from FAME software (Figure 2).

### 3.2. mRNA Expression of Potential Downstream Target Genes of hsa-miR-590-3p

**Figure 3:**
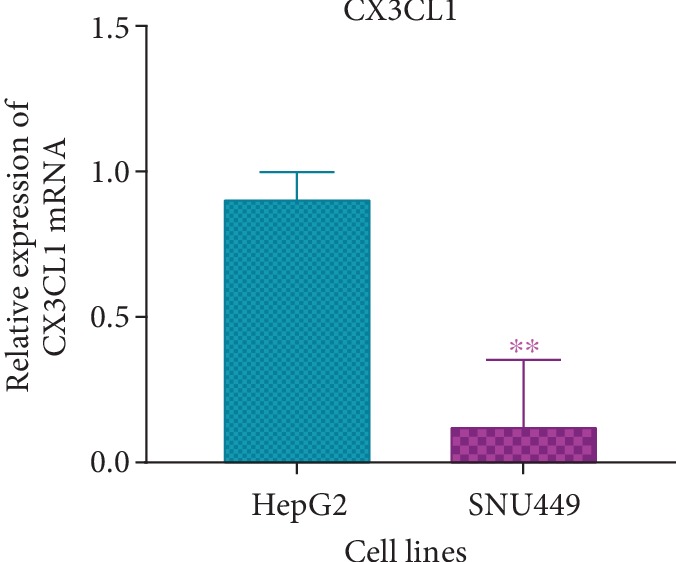
Graphical representation of CX3CL1 mRNA expression in HepG2 and SNU449. RT-PCR band intensities were measured using ImageJ and normalized against GAPDH and statistically analyzed using Prism GraphPad. *P* values were computed using one-way ANOVA (with a Bonferroni posttest). *P* values less than 0.05 are considered significant (^∗^*P* value < 0.05, ^∗∗^*P* value < 0.01, and ^∗∗∗^ *P* value < 0.001). Results are a representation of three independent experiments.

The mRNA levels of the potential target genes of hsa-miR-590-3p were assessed in HepG2 and SNU449 using RT-PCR. mRNA expression levels of all genes showed no significant difference between HepG2 and SNU449 except for CX3CL1 ([Supplementary-material supplementary-material-1]). CX3CL1 mRNA expression was significantly higher in HepG2 compared to SNU449 (*P* < 0.01) (Figure 3, [Supplementary-material supplementary-material-1]).

### 3.3. Alignment of Potential Targets of hsa-miR-590-3p Using miRanda-mirSVR

**Figure 4:**
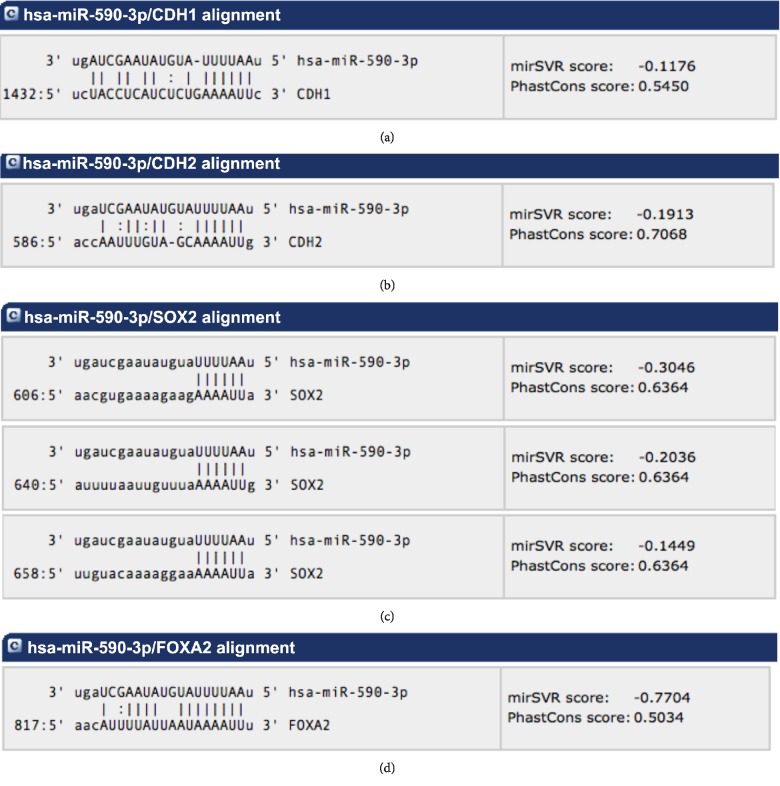
Alignment of hsa-miR-590-3p against the mRNA of E-cadherin, N-cadherin, SOX2, and FOXA2 using miRanda-mirSVR. (a) Binding site of hsa-miR-590-3p on E-cadherin mRNA at position 1432. (b) Binding site of hsa-miR-590-3p on N-cadherin mRNA at position 586. (c) Binding sites of hsa-miR-590-3p on SOX2 mRNA at positions 606, 640, and 658. (d) Binding site of hsa-miR-590-3p on FOXA2 mRNA at position 817.

Through the alignment of hsa-miR-590-3p against the mRNA of some genes using miRanda-mirSVR, more genes were identified as potential downstream target genes of hsa-miR-590-3p. These genes are E-cadherin, N-cadherin, SOX2, and FOXA2.  Figure 4 illustrates the binding site(s) of hsa-miR-590-3p on all four genes. hsa-miR-590-3p has one binding site on E-cadherin mRNA (Figure 4(a)) and one binding site on N-cadherin mRNA (Figure 4(b)), three binding sites on SOX2 mRNA (Figure 4(c)), and one binding site on FOXA2 mRNA (Figure 4(d)).

### 3.4. Protein Expression of Potential Targets of hsa-miR-590-3p Using Expression Atlas

**Figure 5:**
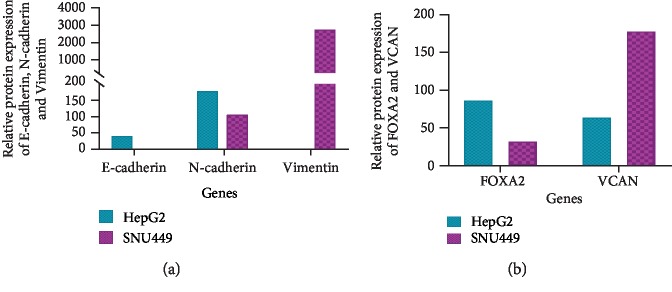
Relative protein expression of potential targets of hsa-miR-590-3p in HCC cell lines using the Expression Atlas. (a) Graphical representation of the relative protein expression of E-cadherin, N-cadherin, and Vimentin in HepG2 and SNU449. (b) Graphical representation of the relative protein expression of FOXA2 and VCAN in HepG2 and SNU449.

The protein expression of E-cadherin, N-cadherin, and FOXA2 was assessed computationally using the Expression Atlas (Figure 5). Also, Vimentin was assessed as an epithelial-mesenchymal transition (EMT) marker and VCAN as a downstream target gene of FOXA2 (Figure 5).

### 3.5. E-Cadherin and N-Cadherin mRNA Expression as Potential Targets of hsa-miR-590-3p Using Vimentin mRNA Expression as a Mesenchymal Marker

**Figure 6:**
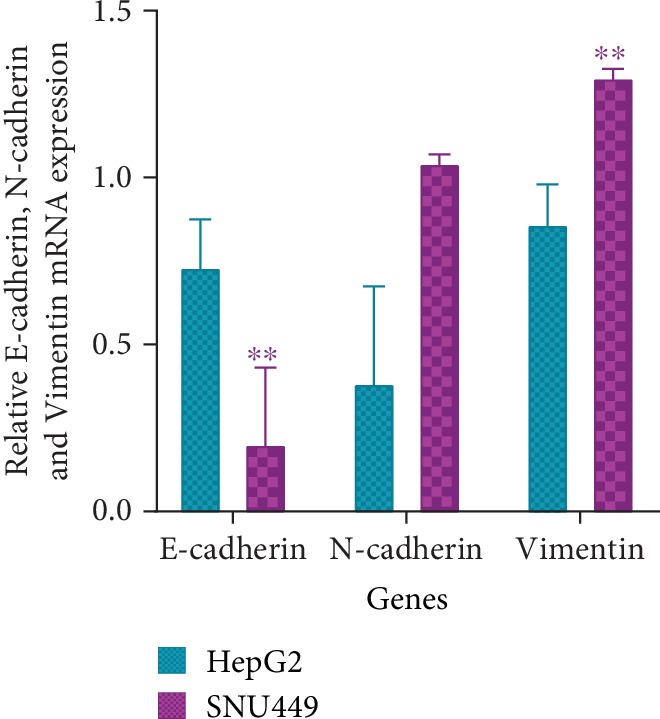
Graphical representation of E-cadherin, N-cadherin, and Vimentin relative mRNA expression in HepG2 and SNU449. RT-PCR band intensities were measured using ImageJ and normalized against GAPDH and statistically analyzed using Prism GraphPad. *P* values were computed using one-way ANOVA (with a Bonferroni posttest). *P* values less than 0.05 are considered significant (^∗^*P* value < 0.05, ^∗∗^*P* value < 0.01, and ^∗∗∗^*P* value < 0.001). Results are a representation of three independent experiments.

E-cadherin, N-cadherin, and Vimentin mRNA levels in HepG2 and SNU449 were analyzed using RT-PCR. E-cadherin mRNA expression was found to be elevated in HepG2 compared to SNU449 (*P* < 0.01) (Figure 6, [Supplementary-material supplementary-material-1]). N-cadherin mRNA expression showed no statistically significant difference between HepG2 and SNU449 (*P* > 0.05) (Figure 6, [Supplementary-material supplementary-material-1]). Vimentin mRNA expression was found to be elevated in SNU449 compared to HepG2 (*P* < 0.01) (Figure 6, [Supplementary-material supplementary-material-1]).

### 3.6. Vimentin Protein Expression as a Mesenchymal Marker

**Figure 7:**
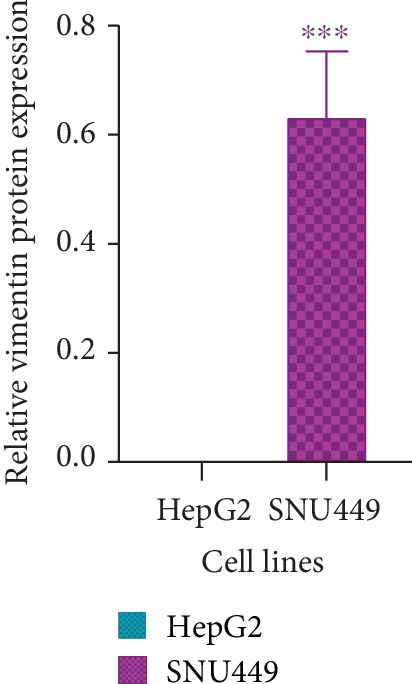
Graphical representation of Vimentin protein expression in HepG2 and SNU449 using western blotting. Western blotting band intensities were measured using ImageJ and normalized against GAPDH and statistically analyzed using Prism GraphPad. *P* values were computed using one-way ANOVA (with a Bonferroni posttest). *P* values less than 0.05 are considered significant (^∗^*P* value < 0.05, ^∗∗^*P* value < 0.01, and ^∗∗∗^*P* value < 0.001). Results are a representation of three independent experiments.

Vimentin protein level in HepG2 and SNU449 was analyzed using western blotting. Vimentin protein is expressed in SNU449 while it is not detected in HepG2 (*P* < 0.001) (Figure 7, [Supplementary-material supplementary-material-1]).

### 3.7. SOX2 mRNA and Protein Expression as a Potential Target of hsa-miR-590-3p

**Figure 8:**
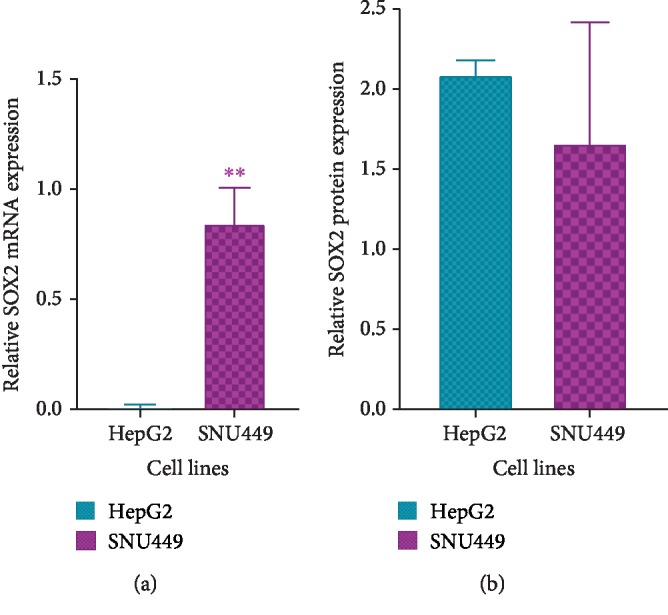
SOX2 mRNA and protein expression in HepG2 and SNU449 using RT-PCR and western blotting, respectively. (a) Graphical representation of SOX2 mRNA expression in HepG2 and SNU449 using RT-PCR. (b) Graphical representation of SOX2 protein expression using western blotting. RT-PCR and western blotting band intensities were measured using ImageJ and normalized against GAPDH and *β*-tubulin, respectively, and statistically analyzed using Prism GraphPad. *P* values were computed using one-way ANOVA (with a Bonferroni posttest). *P* values less than 0.05 are considered significant (^∗^*P* value < 0.05, ^∗∗^*P* value < 0.01, and ^∗∗∗^*P* value < 0.001). Negatives were carried out for RT-PCR experiments. Results are a representation of three independent experiments.

SOX2 mRNA and protein levels in HepG2 and SNU449 were analyzed using RT-PCR and western blotting, respectively. On the mRNA level, SNU449 shows increased SOX2 expression compared to HepG2 (*P* < 0.01) (Figure 8(a), [Supplementary-material supplementary-material-1]). On the protein level, HepG2 and SNU449 show comparable SOX2 expression (*P* > 0.05) (Figure 8(b), [Supplementary-material supplementary-material-1]).

### 3.8. FOXA2 mRNA Expression as a Potential Target of hsa-miR-590-3p and Its Downstream Target Gene VCAN mRNA Expression

**Figure 9:**
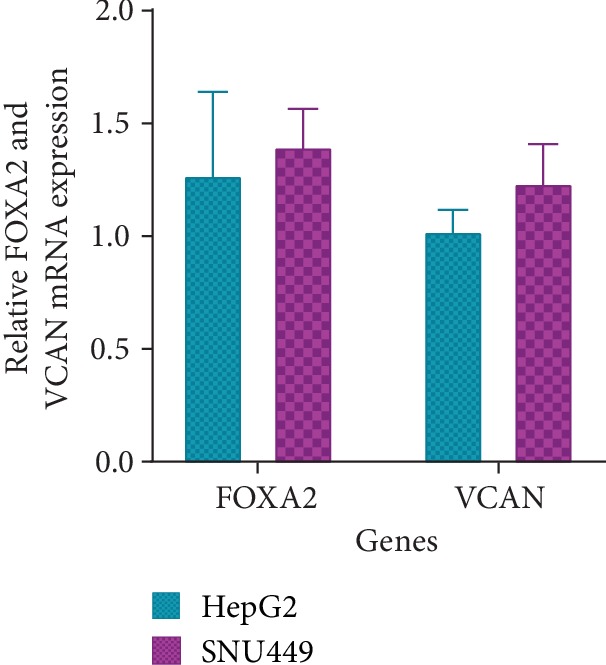
Graphical representation of FOXA2 and VCAN relative mRNA expression in HepG2 and SNu449 using RT-PCR. RT-PCR band intensities were measured using ImageJ and normalized against GAPDH and statistically analyzed using Prism GraphPad. *P* values were computed using one-way ANOVA (with a Bonferroni posttest). *P* values less than 0.05 are considered significant (^∗^*P* value < 0.05, ^∗∗^*P* value < 0.01, and ^∗∗∗^*P* value < 0.001). Negatives were carried out for all experiments. Results are a representation of three independent experiments.

FOXA2 and VCAN mRNA levels in HepG2 and SNU449 were analyzed using RT-PCR. No statistically significant difference was observed between the FOXA2 and VCAN mRNA expression in HepG2 and SNU449 (*P* > 0.05) (Figure 9, [Supplementary-material supplementary-material-1]).

### 3.9. Expression and Localization of hsa-miR-590-3p and Its Potential Target, SOX2, in SNU449 Cells

**Figure 10:**
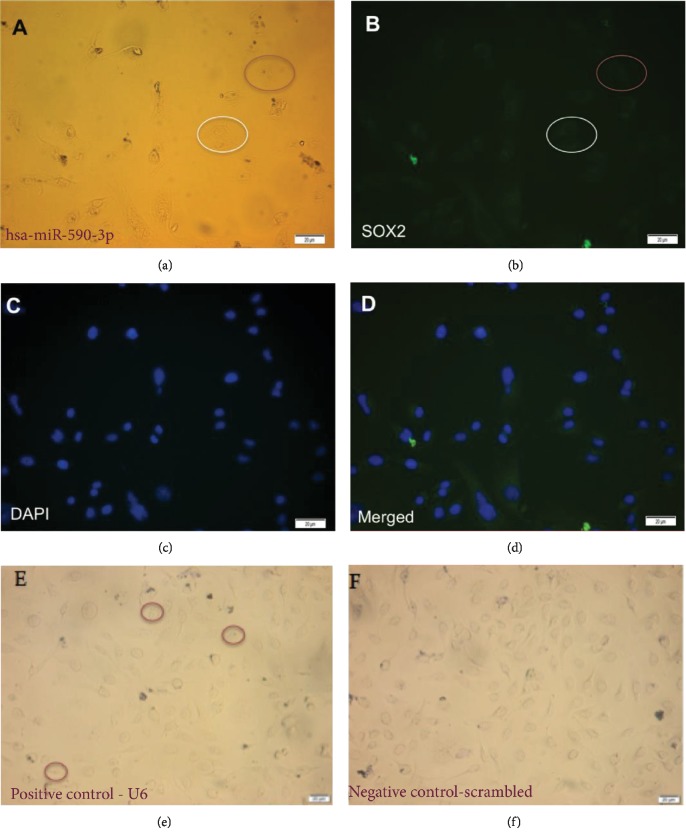
Expression and localization of hsa-miR-590-3p and SOX2 in SNU449 cells. (a) hsa-miR-590-3p signal in SNU449 cells under 20x magnification using ISH (red circle). (b) SOX2 fluorescent signal in SNU449 cells under 20x magnification using ICC (white circle). (c) DAPI staining of the nucleus. (d) Merged image of (b) and (c). (e) U6 signal SNU449 cells under 20x magnification using ISH as a positive control (red circles). (f) Negative control in SNU449 cells under 20x magnification using ISH using scrambled probes. Scale for (a)–(f): 20 *μ*m.

**Figure 11:**
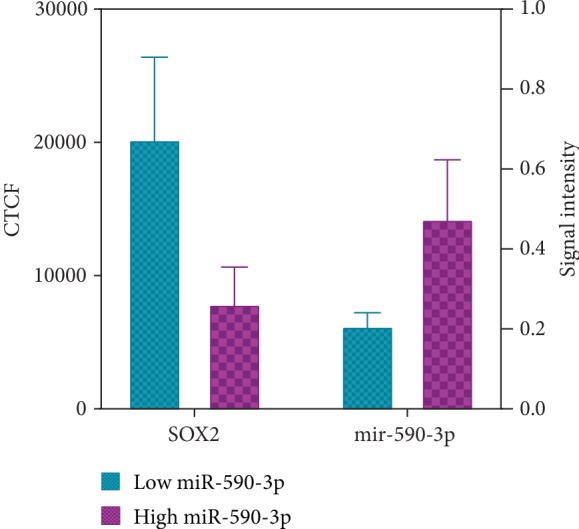
Graphical representation of the expression of hsa-miR-590-3p and SOX2 in SNU449 cells. The cell fluorescence of SOX2 and the signal intensity of hsa-miR-590-3p were assessed using ImageJ and compared using Prism GraphPad. Low-expressing hsa-miR-590-3p cells show increased SOX2-corrected total cell fluorescence (CTCF) compared to high-expressing has-miR-590-3p cells, which show low SOX2 CTCF. Results are a representation of three independent experiments.

Expression and localization of hsa-miR-590-3p and SOX2 in SNU449 cells were assessed using ISH-ICC (Figures 10 and 11). hsa-miR-590-3p signal was detected in the cytoplasm of SNU449 cells. SOX2 fluorescence was detected mainly in the cytoplasm of the SNU449 cells. Cells that showed hsa-miR-590-3p signal showed minimal SOX2 fluorescence (red circles, Figure 10), and in cells that did not show hsa-miR-590-3p signal, SOX2 fluorescence was detected (white circles, Figure 10).

## 4. Discussion

Hepatocellular carcinoma is an aggressive malignant tumor with poor prognosis. It is usually diagnosed during the late stages of the cancer when most medications and surgical interventions are not efficient. Hence, understanding the cancer progression and pathogenesis is important.

miRNAs were discovered twenty years ago, broadening our understanding of cancer pathogenesis. Several miRNAs have been studied previously in relation to HCC and different liver disease including HCV, HBV, and non-alcoholic fatty liver disease. These miRNAs include miR-17, miR-21, miR-22, miR-26, miR-29b, miR-122, miR-135a, miR-146a, miR-151, miR-181b, miR-221/222, miR-224, miR-233, miR-338-3p, miR-491, and miR-500 [8, 9, 25–28]. They were found in the serum and liver tissues of HCC patients; some were found to be upregulated while others were found to be downregulated.

Several studies tested hsa-miR-590-3p in relation to various cancers. In glioblastoma multiforme (GBM), an aggressive brain cancer, hsa-miR-590-3p was significantly downregulated in cancer tissue compared to normal tissues [29], while in epithelial ovarian cancer (EOC) hsa-miR-590-3p was significantly upregulated in cancer tissue compared to that of the normal ovarian tissues [30]. This difference in expression in two different cancers suggests that hsa-miR-590-3p is tissue-specific and can regulate tumor suppressor genes and oncogenes in different tissues.

hsa-miR-590-3p was also reported to be expressed in HCC by two studies. In the first study, hsa-miR-590-3p was significantly downregulated in cancer tissue compared to normal tissues [31], while in the second study, it was reported to be significantly upregulated in three HCC cell lines (HepG2, Hep3B, and Huh7) [32]. The inconsistency of the reported findings may result from the fact that tissue specimens are heterogeneous and that cell lines may change their characteristics and in turn their gene expression when they are in culture for a long time.

### 4.1. Prediction of Potential Downstream Target Genes of hsa-miR-590-3p

In this study, several bioinformatics analyses were carried out to predict potential downstream target genes of hsa-miR-590-3p. First, preliminary screening of five databases (TargetScan, miRanda-mirSVR, miRDB, miRTarBase, and Diana Tools) was carried out ([Supplementary-material supplementary-material-1]), giving rise to a list of potential downstream target genes. Second, the FAME software was used for the function prediction approach ([Supplementary-material supplementary-material-1]), giving rise to a list of potential downstream target genes based on function. Thirty-two genes from the first approach were found in the list from the second approach. Through the usage of previous literature and The Human Protein Atlas to assess protein levels of the potential targets of hsa-miR-590-3p in normal liver tissue versus cancerous liver tissue, fourteen genes were chosen for further validation using molecular techniques (Figure 1).

### 4.2. Potential Downstream Target Genes of hsa-miR-590-3p

The fourteen genes are BRIP1, CX3CL1, DCLRE1A, DLG1, DYRK2, ERCC5, FANCF, HIPK2, MLH3, NPHP1, RAD21, SMC6, TMEM33, and UVRAG. Figure 2 categorizes the genes according to the functions they share.

UVRAG, SMC6, MLH3, FANCF, and RAD21 belong to the first category: response to DNA damage stimulus and DNA repair. UVRAG encodes the UV radiation resistance-associated protein, which activates the Beclin1-PI(3)KC3 complex that promotes autophagy and inhibits the proliferation and tumorigenicity of human colon cancer cells [33]. SMC6 encodes the structural maintenance of chromosome 6 protein and is mostly involved in DNA repair [34]. MLH3 is a member of the MutL-homolog family that is involved with DNA mismatch repair genes [35]. FANCF, known as Fanconi anemia complementation group F, are essential in DNA repair [36]. The RAD21 cohesin complex component is important for mitotic growth and has a role in the repair of DNA double-strand breaks [37].

BRIP1, DYRK2, and HIPK2 belong to the second category: response to DNA damage stimulus and DNA damage response and signal transduction. BRIP1, known as BRCA1-interacting protein C-terminal helicase 1, forms a complex with BRCA1 that is essential in the repair of double-strand breaks [38]. DYRK2, known as dual specificity tyrosine phosphorylation-regulated kinase 2, is part of a protein kinase family which is involved in cellular growth and development [39]. HIPK2, known as homeodomain-interacting protein kinase 2, is a cell growth and apoptosis regulator [40].

ERCC5 and DCLRE1A belong to the third category: response to DNA damage stimulus, DNA repair, and nucleotide excision repair. ERCC5, or excision repair cross-complementing 5, encodes a single-strand-specific DNA endonuclease that creates a 3′ incision following UV-induced damage in the DNA excision repair [41]. DCLRE1A, or DNA cross-link repair 1A, encodes a conserved protein that has a role in the DNA interstrand cross-link repair [42].

TMEM33, or transmembrane protein 33, is a transmembrane protein involved in endoplasmic reticulum stress-responsive events in cancer cells [43]. CX3CL1, DLG1, and NPHP1 are involved in cell-cell adhesion. DLG1, or human discs large tumor suppressor, regulates cell polarity and proliferation suggesting a connection between epithelial organization and cellular growth control [44]. NPHP1, nephrocystin 1, encodes a protein that interacts with a Crk-associated substrate, and it is involved in cell division and cell-cell and cell-matrix adhesion [45]. CX3CL1, or C-X3-C motif chemokine ligand 1 or chemokine fractalkine, has been reported in many epithelial tissues and facilitates strong cell adhesion [46].

The mRNA expression of all these genes was assessed in the HCC cell lines, HepG2 and SNU449. All the genes showed comparable expression between both cell lines except CX3CL1 ([Supplementary-material supplementary-material-1]). HepG2 showed high CX3CL1 mRNA expression compared to SNU449 (Figure 3, [Supplementary-material supplementary-material-1]). HepG2 is an early-stage well-differentiated human HCC cell line while SNU449 is an intermediate stage hepatitis B virus- (HBV-) infected HCC cell line. This explains the obtained results as high expression of CX3CL1 indicates tighter cell adhesion, thus no metastasis.

Since CX3CL1 is the only gene that showed differential expression between the tested HCC cell lines and it is involved in cell-cell adhesion, we decided to choose genes related to that function and validate them as potential downstream target genes of hsa-miR-590-3p but using miRanda-mirSVR. E-cadherin and N-cadherin mRNA were aligned against hsa-miR-590-3p to assess the binding sites of our miRNA on the mRNA of these genes (Figures 4(a) and 4(b)).

### 4.3. Potential Targets of hsa-miR-590-3p and Cell-Cell Adhesion

Calcium-dependent cell adhesion molecules known as cadherins are mainly involved in cell-cell adhesion and cell migration [47]. There are several types of cadherins, but in our study, we are focusing on E-cadherin (CDH1) and N-cadherin (CDH2). E-cadherin, or epithelial cadherin, is the protein that holds epithelial cells together. As cancer progresses, cells start to lose E-cadherin expression and start producing N-cadherin. N-cadherin, or neural cadherin, increases with the cell's increased invasiveness potential [48]. These molecular changes take place through a well-studied phenomenon known as epithelial-mesenchymal transition (EMT). EMT is a process in which epithelial cells lose their adherent nature and acquire mesenchymal traits, including migration and invasion abilities hence the ability to metastasize to other organs [49]. These molecular changes include alteration in Vimentin expression (a mesenchymal marker), which increases as the cancer progresses and the cells become more invasive [47].

E-cadherin, N-cadherin, and Vimentin protein expression in HepG2 and SNU449 were assessed computationally using the Expression Atlas. E-cadherin and N-cadherin protein expression is higher in HepG2 compared to SNU449 while Vimentin protein is expressed in SNU449 and not detected in HepG2 (Figure 5).

E-cadherin, N-cadherin, and Vimentin mRNA expression was assessed in HepG2 and SNU449 using RT-PCR (Figure 6, [Supplementary-material supplementary-material-1]). HepG2 showed high E-cadherin expression, low N-cadherin expression, and low Vimentin expression compared to SNU449. Since HepG2 is of epithelial origin, it is retaining its epithelial characteristics, hence the increase in E-cadherin, an epithelial marker, and the decrease in N-cadherin and Vimentin, mesenchymal markers. SNU449 showed low E-cadherin expression, high N-cadherin expression, and high Vimentin expression compared to HepG2. Since SNU449 is at a more advanced stage of the cancer, it acquired some mesenchymal characteristics, hence the decrease in the epithelial marker, E-cadherin, and the increase in the mesenchymal markers, N-cadherin and Vimentin.

Vimentin protein expression was assessed in HepG2 and SNU449 using western blotting (Figure 7, [Supplementary-material supplementary-material-1]). Vimentin protein expression was highly expressed in SNU449 while it was not detected in HepG2. Two bands were observed in SNU449; the second band could be a result of alterative splicing or posttranslational modification [50, 51]. As for HepG2, the protein is not detected while the mRNA is expressed. This can be attributed to the degradation of the mRNA or inhibition of translation. Our Vimentin protein expression results of both cell lines match our computational results via the Expression Atlas.

### 4.4. Potential Targets of hsa-miR-590-3p and the FOXA2-VCAN Pathway

In a previous study, FOXA2 was identified as a potential downstream target gene of miR-590-3p in epithelial ovarian cancer (EOC) [30]. Forkhead box A2 (FOXA2) is part of the FOXA family. It is a transcription factor that is associated with embryo development regulation and metabolism and homeostasis during the adult stage. Its involvement in hepatic specification and its importance for hepatic glucose and lipid homeostasis were previously reported in a study [52]. FOXA2's dual role in cancer development as a tumor suppressor and a tumor promoter in various cancers has been reported by several studies [30, 52]. Interestingly, it was also found to be sexually dimorphic in HCC; meaning it is tumor suppressing in females and tumor promoting in males [52].

A recent study proposes that VCAN is a downstream target gene of FOXA2 and shows that there is a negative correlation between their mRNA expression in EOC [30]. Versican (VCAN) is part of the aggrecan/versican proteoglycan family and a main component of the extracellular matrix. Its important role in tumor development through cell adhesion, proliferation, migration, invasion, and angiogenesis has been reported in several studies [30, 53].

Alignment of the mRNA of FOXA2 against hsa-miR-590-3p to assess the binding sites of our miRNA on its mRNA was carried out using miRanda-mirSVR (Figure 4(d)). FOXA2 and VCAN protein expression in HepG2 and SNU449 was assessed computationally using the Expression Atlas. FOXA2 protein expression is higher in HepG2 compared to SNU449 while VCAN protein expression is higher in SNU449 compared to HepG2 (Figure 5).

FOXA2 and VCAN mRNA expression were assessed in HepG2 and SNU449 using RT-PCR (Figure 9, [Supplementary-material supplementary-material-1]). No statistically significant change was observed among the mRNA levels of FOXA2 and VCAN in both cell lines.

### 4.5. Potential Downstream Target Genes of miR-590-3p and Cell Stemness

SOX2 also has been previously reported as a target of hsa-miR-590-3p in EOC [54]. SOX2, known as SRY (sex-determining region on the Y chromosome) box 2, is part of the SOX family and is important in reprogramming differentiated cells into induced pluripotent stem cells and maintaining cell self-renewal [55]. It was also reported to participate in oncogenesis and tumor progression of several cancers.

Alignment of the mRNA of SOX2 against hsa-miR-590-3p to assess the binding sites of our miRNA on its mRNA was carried out using miRanda-mirSVR (Figure 4(c)). SOX2 mRNA and protein expression were assessed in HepG2 and SNU449 using RT-PCR and western blotting, respectively (Figure 8, [Supplementary-material supplementary-material-1]). SNU449 showed higher mRNA expression than HepG2. Surprisingly, HepG2 and SNU449 showed comparable protein expression. Since HepG2 is an early-stage well-differentiated cell line, we expected that SOX2 protein levels in HepG2 would be lower than SNU449.

### 4.6. Expression and Localization of hsa-miR-590-3p and SOX2

The co-expression and co-localization of hsa-miR-590-3p in relation to one of its downstream target genes, SOX2, in SNU449 were carried out using ISH-ICC. A negative correlation between the hsa-miR-590-3p expression and the SOX2 expression was observed. Cells that showed hsa-miR-590-3p signal showed minimal SOX2 fluorescence (red circles, Figures 10 and 11), and in cells that did not show hsa-miR-590-3p signal, SOX2 fluorescence was detected (white circles, Figures 10 and 11). These findings strongly suggest that SOX2 can be a direct downstream target gene of hsa-miR-590-3p in HCC. However, it remains to be determined whether the consequences of overexpressing hsa-miR-590-3p suppresses the expression of the 3′UTR of SOX2, utilizing the dual-luciferase reporter assay.

## 5. Conclusion

In conclusion, our study suggests that SOX2 can be a direct downstream target gene of hsa-miR-590-3p in HCC implying that hsa-miR-590-3p can directly affect the self-renewal and self-maintenance of cancer cells. We propose that CX3CL1, E-cadherin, N-cadherin, and FOXA2 show a lot of potential as downstream target genes of hsa-miR-590-3p signifying hsa-miR-590-3p's indirect effect on EMT and in turn cancer progression. Nevertheless, more studies are needed to further prove our work.

## Data Availability

The data used to support the findings of this study are included within the article and within the supplementary files.

## Supplementary Materials

Supplementary Materials Supplementary A: primers used in semiquantitative RT-PCR. Supplementary B: downstream target genes of hsa-miR-590-3p obtained from TargetScan. Supplementary C: downstream target genes of hsa-miR-590-3p obtained from miRDB. Supplementary D: downstream target genes of hsa-miR-590-3p obtained from miRTarBase. Supplementary E: downstream target genes of hsa-miR-590-3p obtained from Diana Tools. Supplementary F: pivot table. Supplementary G: the chosen functions of the potential downstream target genes of hsa-miR-590-3p obtained from FAME Software. Supplementary H1: mRNA expression of potential targets of hsa-miR-590-3p in HepG2 and SNU449 using RT-PCR. Supplementary H2: RT-PCR analysis for CX3CL1 mRNA expression in HepG2 and SNU449. Supplementary H3: RT-PCR analysis for E-cadherin, N-cadherin, and Vimentin mRNA expression in HepG2 and SNU449. Supplementary H4: membrane image for Vimentin protein expression in HepG2 and SNU449. Supplementary H5: SOX2 mRNA and protein expression in HepG2 and SNU449 using RT-PCR and western blotting. Supplementary H6: RT-PCR analysis for FOXA2 and VCAN mRNA expression in HepG2 and SNU449.
