# Supplementary material for: miR-590-3p and Its Downstream Target Genes in HCC Cell Lines
Source: Anal Cell Pathol (Amst). 2019 Nov 3;2019:3234812. doi: 10.1155/2019/3234812 (PMC6875279; doi:10.1155/2019/3234812)
Supplement: Supplementary Materials — Supplementary A: primers used in semiquantitative RT-PCR. Supplementary B: downstream target genes of hsa-miR-590-3p obtained from TargetScan. Supplementary C: downstream target genes of hsa-miR-590-3p obtained from miRDB. Supplementary D: downstream target genes of hsa-miR-590-3p obtained from miRTarBase. Supplementary E: downstream target genes of hsa-miR-590-3p obtained from Diana Tools. Supplementary F: pivot table. Supplementary G: the chosen functions of the potential downstream target genes of hsa-miR-590-3p obtained from FAME Software. Supplementary H1: mRNA expression of potential targets of hsa-miR-590-3p in HepG2 and SNU449 using RT-PCR. Supplementary H2: RT-PCR analysis for CX3CL1 mRNA expression in HepG2 and SNU449. Supplementary H3: RT-PCR analysis for E-cadherin, N-cadherin, and Vimentin mRNA expression in HepG2 and SNU449. Supplementary H4: membrane image for Vimentin protein expression in HepG2 and SNU449. Supplementary H5: SOX2 mRNA and protein expression in HepG2 and SNU449 using RT-PCR and western blotting. Supplementary H6: RT-PCR analysis for FOXA2 and VCAN mRNA expression in HepG2 and SNU449. [file 3234812.f1.zip › Supplementary A (1).docx]

**Supplementary A. Primers used in Semi-quantitative RT-PCR.**

| **Genes** | **Primer Sequence** | **Number of cycles** | **Annealing Temperature (°C)** | **Amplicon Size (bp)** |
| --- | --- | --- | --- | --- |
| GAPDH | F: 5’-CCACCCATGGCAAATTCCATGGCA-3’  R: 5’-TCTAGACGGCAGGTCAGGTCCACC-3’ | 27 | 60.5 | 598 |
| SOX2 | F: 5'-TTACCTCTTCCTCCCACTCC-3'  R: 5'-CCTCCCATTTCCCTCGTTT-3' | 35 | 57 | 252 |
| E-cadherin | F: 5’-CCTTCCTCCCAATACATCTCCC-3’  R: 5’-TCTCCGCCTCCTTCTTCATC-3’ | 30 | 58 | 432 |
| N-cadherin | F: 5’-CACTGCTCAGGACCCAGAT-3’  R: 5’-TAAGCCGAGTGATGGTCC-3’ | 30 | 60 | 416 |
| Vimentin | F: 5’-GAACGCCAGATGCGTGAAATG-3’  R: 5’- CCAGAGGGAGTGAATCCAGATTA -3’ | 35 | 60 | 280 |
| FOXA2 | F: 5'-TCTTGACACGGTGAAATCCA-3'  R: 5'-CCTGCAACCAGACAGGGTAT-3' | 40 | 55 | 270 |
| VCAN | F: 5′GTGACTATGGCTGGCACAAATTCC-3′  R: 5′-GGTTGGGTCTCCAATTCTCGTATTGC- 3’ | 35 | 55 | 255 |
| TMEM33 | F: 5’-GTTGTGGGGGAATAATGTGC-3’  R: 5’-TTCCAATCATGCAACGGTAA-3’ | 30 | 54 | 172 |
| DCLRE1A | F: 5’-AACATGTTCGTGGTGCTGAA-3’  R: 5’-CCTGAATCTGCCTTTGCTTC-3’ | 35 | 57 | 150 |
| ERCC5 | F: 5’-CAGACACAGCTCCGAATTGA-3’  R: 5’-TTCTGGGTTTTTCGTTTTGC-3’ | 35 | 54 | 209 |
| UVRAG | F: 5’-GAGCCCTGCTCTTGAATGTC-3’  R: 5’-TCATGGCAGAAACTGCAGAC-3’ | 35 | 58 | 179 |
| NPHP1 | F: 5’-TGCCAAAGGAAATGAAGGTC-3’  R: 5’-CACTCCAGTGGGGATCAGTT-3’ | 35 | 52 | 167 |
| SMC6 | F: 5’-AGAGCCTTGTCTGGAGGTGA-3’  R: 5’-TCATGCTTTGAGGTGTGAGC-3’ | 30 | 56 | 208 |
| DLG1 | F: 5’-CCAGGCAGGTTACACCAGAT-3’  R: 5’-TCCTGCTCACTCTGGTCCTT-3’ | 30 | 57 | 208 & 211 |
| CX3CL1 | F: 5’-TCTGCCATCTGACTGTCCTG-3’  R: 5’-ACCACAGACTCGTCCATTCC-3’ | 32 | 57 | 213 & 334 |
| BRIP1 | F: 5’-AATACCGAAGGCAACACCTG-3’  R: 5’-CAGAACAGAGCGGATGTTCA-3’ | 32 | 55 | 242 |
| HIPK2 | F: 5’-CACTGTGCAGTCTCCCTCAA-3’  R: 5’-TGAGGCCTGGACTAAGCTGT-3’ | 28 | 57 | 191 |
| DYRK2 | F: 5’-TTCACAAGAGCTGCCACATC-3’  R: 5’-CCCCAAACAATGGTAACCTG-3’ | 35 | 55 | 194 |
| MLH3 | F: 5’-CAGTGCCTGGCATTCTCAC-3’  R: 5’-GAAATGGAAGGGTGCATGAG-3’ | 30 | 57 | 592 |
| FANCF | F: 5’-CGCTTCAATGGCTATAGAGAGAA-3’  R: 5’-GCGGCTAGTCACTAAAGTCAAAA-3’ | 30 | 60 | 360 |
| RAD21 | F: 5′-TGGGTTGTGTTTGTGTTCTG-3′  R: 5′-TCAAGAGGGTGACCATTGTT-3′ | 30 | 57 | 334 |

*F: Forward * R: Reverse *bp: Basepair
