# Supplementary material for: miR-590-3p and Its Downstream Target Genes in HCC Cell Lines
Source: Anal Cell Pathol (Amst). 2019 Nov 3;2019:3234812. doi: 10.1155/2019/3234812 (PMC6875279; doi:10.1155/2019/3234812)
Supplement: Supplementary Materials — Supplementary A: primers used in semiquantitative RT-PCR. Supplementary B: downstream target genes of hsa-miR-590-3p obtained from TargetScan. Supplementary C: downstream target genes of hsa-miR-590-3p obtained from miRDB. Supplementary D: downstream target genes of hsa-miR-590-3p obtained from miRTarBase. Supplementary E: downstream target genes of hsa-miR-590-3p obtained from Diana Tools. Supplementary F: pivot table. Supplementary G: the chosen functions of the potential downstream target genes of hsa-miR-590-3p obtained from FAME Software. Supplementary H1: mRNA expression of potential targets of hsa-miR-590-3p in HepG2 and SNU449 using RT-PCR. Supplementary H2: RT-PCR analysis for CX3CL1 mRNA expression in HepG2 and SNU449. Supplementary H3: RT-PCR analysis for E-cadherin, N-cadherin, and Vimentin mRNA expression in HepG2 and SNU449. Supplementary H4: membrane image for Vimentin protein expression in HepG2 and SNU449. Supplementary H5: SOX2 mRNA and protein expression in HepG2 and SNU449 using RT-PCR and western blotting. Supplementary H6: RT-PCR analysis for FOXA2 and VCAN mRNA expression in HepG2 and SNU449. [file 3234812.f1.zip › Supplementary G.docx]

**Supplementary G. The Chosen Functions of the Potential Downstream Target Genes of hsa-miR-590-3p obtained from FAME Software.**

| **Function** | **FAME p-value** | **FAME Enrichment factor** | **Genes linked to Function** |
| --- | --- | --- | --- |
| Response to DNA Damage Stimulus | 0.0198 | 1.51 | ANKRD17, BRIP1, CHES1, DCLRE1A, DYRK2, EPC2, ERCC5, ESCO2, FANCF, HIPK2, MAPK1, MLH3, MYO6, POLS, RAD21, RAD23B, SFPQ, SMC6, UBE2N and UVRAG |
| DNA Repair | 0.0251 | 1.62 | ANKRD17, BRIP1, CHES1, DCLRE1A, DYRK2, EPC2, ERCC5, ESCO2, FANCF, MLH3, POLS, RAD21, RAD23B, SFPQ, SMC6, UBE2N and UVRAG |
| Cell-cell Adhesion | 0.0361 | 1.5 | CD164, CLDN22, CLSTN1, CTNND2, CX3CL1, DLG1, DSC3, FAT3, MSN, NLGN1, NPHP1, PCDH19, PKHD1 and VCAM1 |
| Nucleotide-Excision Repair | 0.0382 | 3.01 | DCLRE1A, ERCC5 and RAD23B |
| DNA Damage Response and Signal Transduction | 0.0439 | 1.81 | BRIP1, CHES1, DYRK2, HIPK2 and MYO6 |
