# Supplementary material for: miR-590-3p and Its Downstream Target Genes in HCC Cell Lines
Source: Anal Cell Pathol (Amst). 2019 Nov 3;2019:3234812. doi: 10.1155/2019/3234812 (PMC6875279; doi:10.1155/2019/3234812)
Supplement: Supplementary Materials — Supplementary A: primers used in semiquantitative RT-PCR. Supplementary B: downstream target genes of hsa-miR-590-3p obtained from TargetScan. Supplementary C: downstream target genes of hsa-miR-590-3p obtained from miRDB. Supplementary D: downstream target genes of hsa-miR-590-3p obtained from miRTarBase. Supplementary E: downstream target genes of hsa-miR-590-3p obtained from Diana Tools. Supplementary F: pivot table. Supplementary G: the chosen functions of the potential downstream target genes of hsa-miR-590-3p obtained from FAME Software. Supplementary H1: mRNA expression of potential targets of hsa-miR-590-3p in HepG2 and SNU449 using RT-PCR. Supplementary H2: RT-PCR analysis for CX3CL1 mRNA expression in HepG2 and SNU449. Supplementary H3: RT-PCR analysis for E-cadherin, N-cadherin, and Vimentin mRNA expression in HepG2 and SNU449. Supplementary H4: membrane image for Vimentin protein expression in HepG2 and SNU449. Supplementary H5: SOX2 mRNA and protein expression in HepG2 and SNU449 using RT-PCR and western blotting. Supplementary H6: RT-PCR analysis for FOXA2 and VCAN mRNA expression in HepG2 and SNU449. [file 3234812.f1.zip › Supplementary H (3).docx]

**A**

**HepG2 SNU449**


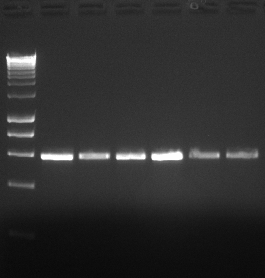


**GAPDH**

**598 bp**


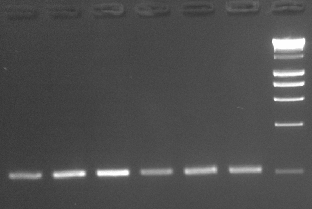


**SMC6**

**208 bp**


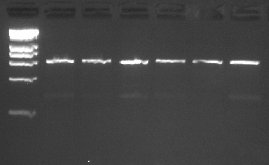


**MLH3**

**592 bp**


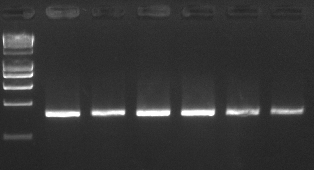


**RAD21**

**334 bp**


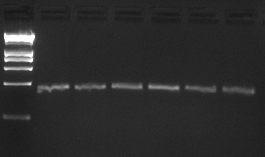


**FANCF**

**360 bp**


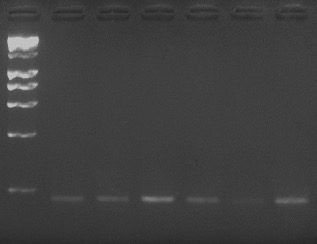


**UVRAG**

**179 bp**

**C**

**B**


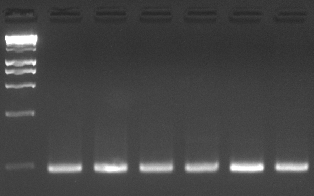


**DYRK2**

**194 bp**


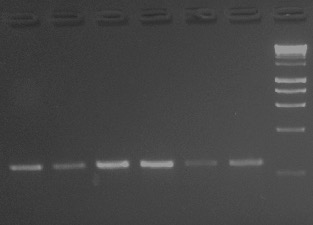


**BRIP1**

**242 bp**


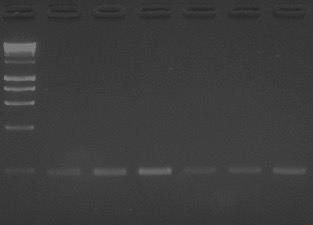


**HIPK2**

**191 bp**

**HepG2 SNU449**


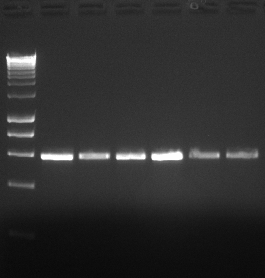


**GAPDH**

**598 bp**


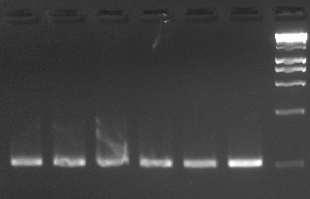


**ERCC5**

**209 bp**


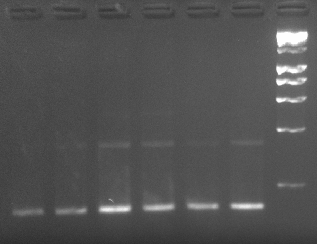


**DCLRE1A**

**150 bp**

**HepG2 SNU449**


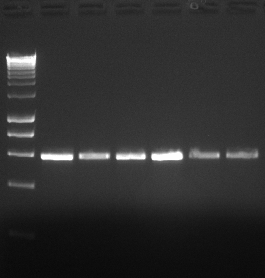


**GAPDH**

**598 bp**

**D**

**E**


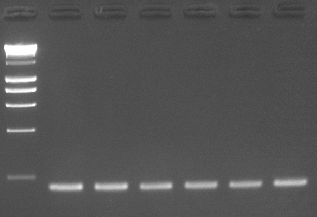


**TMEM33**

**172 bp**

**HepG2 SNU449**


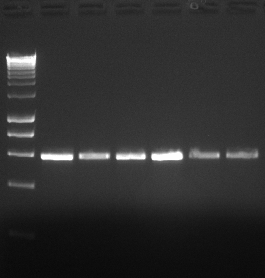


**GAPDH**

**598 bp**


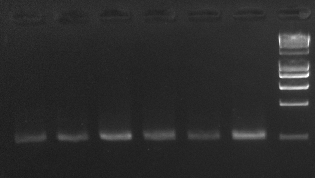


**DLG1**

**208 & 211 bp**


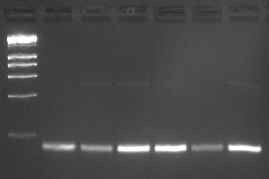


**NPHP1**

**167 bp**

**HepG2 SNU449**


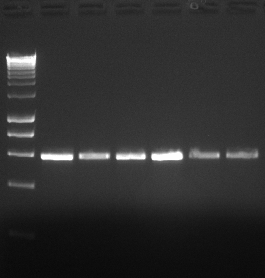


**GAPDH**

**598 bp**


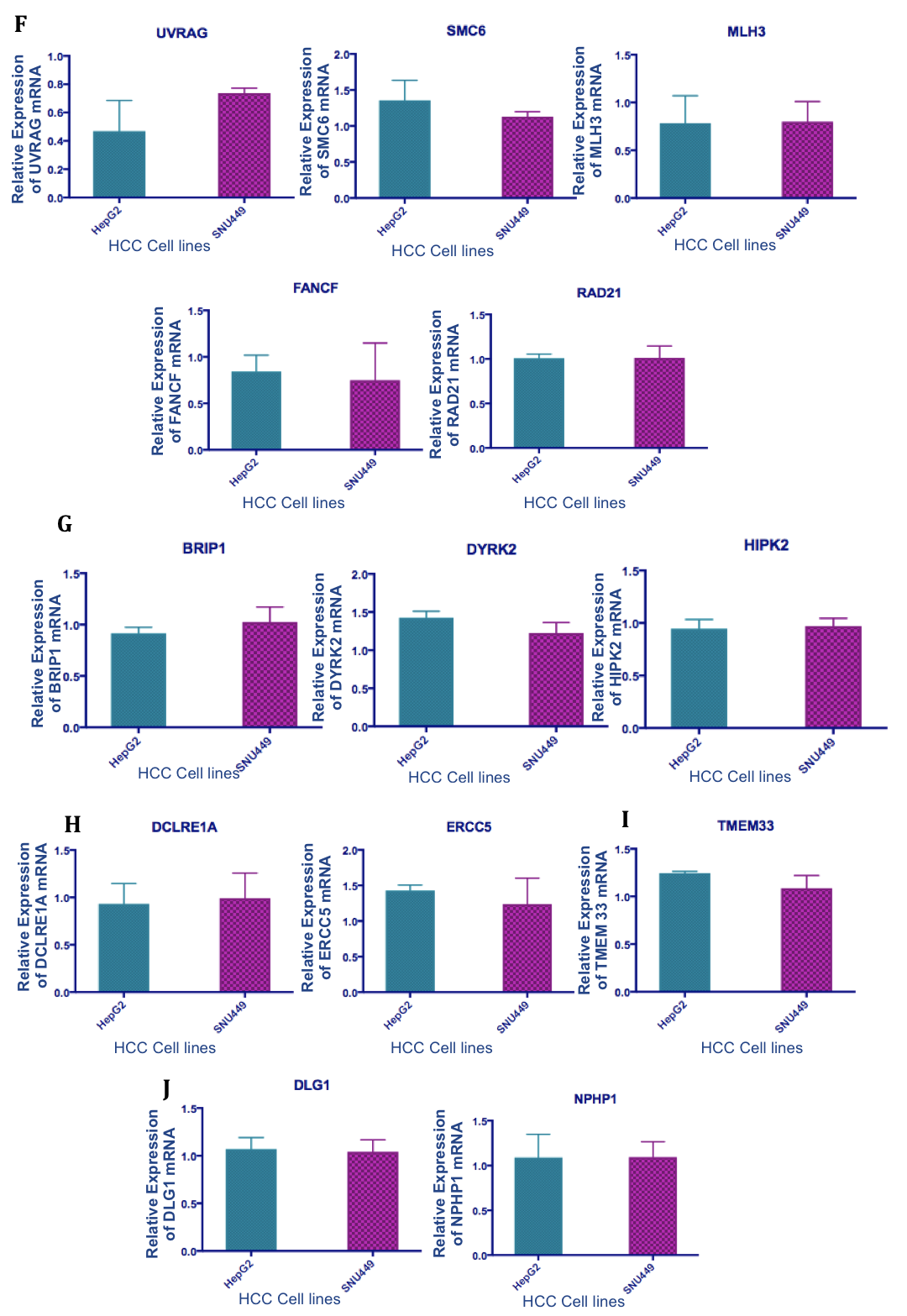


**Supplementary H1**. mRNA Expression of Potential Targets of hsa-miR-590-3p in HepG2 and SNU449 using RT-PCR. A-E. RT-PCR analysis for mRNA expression of potential targets of hsa-miR-590-3p in HepG2 and SNU449. F-J. Graphical representation of mRNA expression of potential targets of hsa-miR-590-3p in HepG2 and SNU449. Images are categorized according to the function of the genes. GAPDH is used as an endogenous control for all genes. Negatives were carried out for all experiments. A&F. Functions: Response to DNA damage stimulus and DNA repair. mRNA expression of UVRAG, SMC6, MLH3, FANCF and RAD21. B&G. Functions: Response to DNA damage stimulus and DNA damage response and signal transduction. mRNA expression of BRIP1, DYRK2 and HIPK2. C&H. Functions: Response to DNA damage stimulus, DNA Repair and nucleotide-excision repair. mRNA expression of ERCC5 and DCLRE1A. D&I. mRNA expression of TMEM33, a trans-membrane protein. E&J. Functions: Cell-cell adhesion. mRNA expression of DLG1 and NPHP1. No statistically significant difference was observed in all genes between both cell lines. RT-PCR band intensities were measured using Image J and normalized against GAPDH and statistically analyzed using Prism GraphPad. P-values were computed using one-way ANOVA (with Bonferroni post-test). P-values less than 0.05 are considered significant (* P-value <0.05, ** P-value <0.01 and *** P-value <0.001). Results are a representation of three independent experiments.


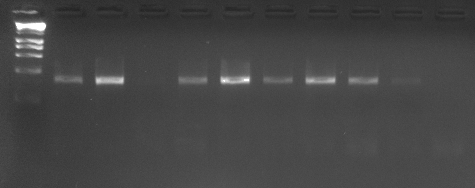


**CX3CL1**

**334 bp**

**HepG2 SNU449**


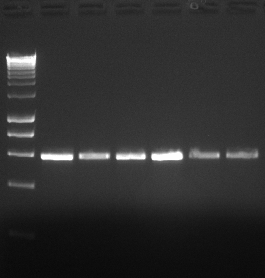


**GAPDH**

**598 bp**

**Supplementary H2**. RT-PCR analysis for CX3CL1 mRNA expression in HepG2 and SNU449. GAPDH was used as an endogenous control. Negatives were carried out for all experiments. Only one amplicon size at 334bp was detected.

**HepG2 SNU449**


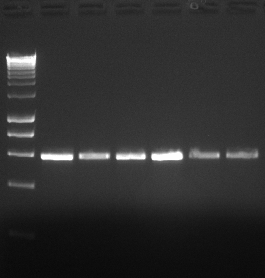


**GAPDH**


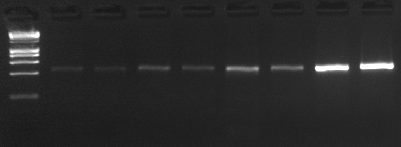


**E-cadherin**

**598 bp**

**432 bp**


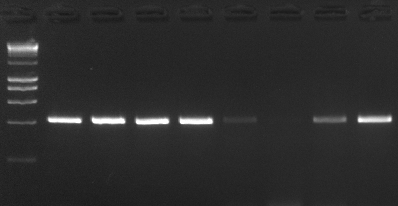


**N-cadherin**

**416 bp**

**Vimentin**


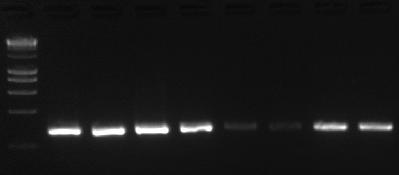

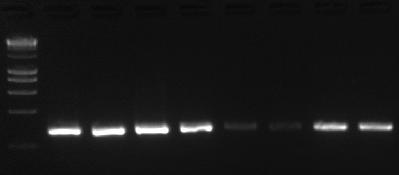


**280 bp**

**Supplementary H3.** RT-PCR analysis for E-cadherin, N-cadherin and Vimentin mRNA expression in HepG2 and SNU449. Negatives were carried out for all experiments.


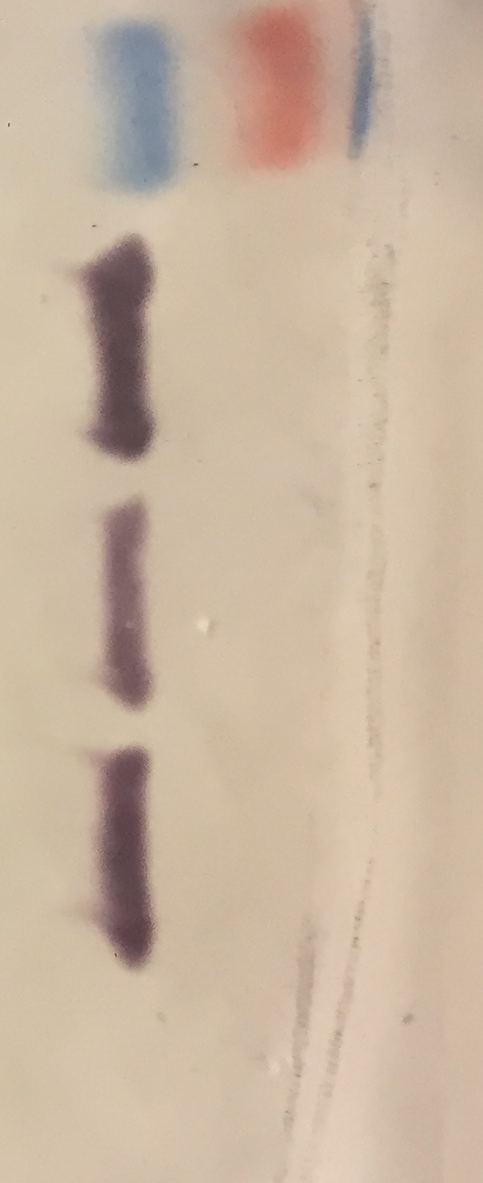

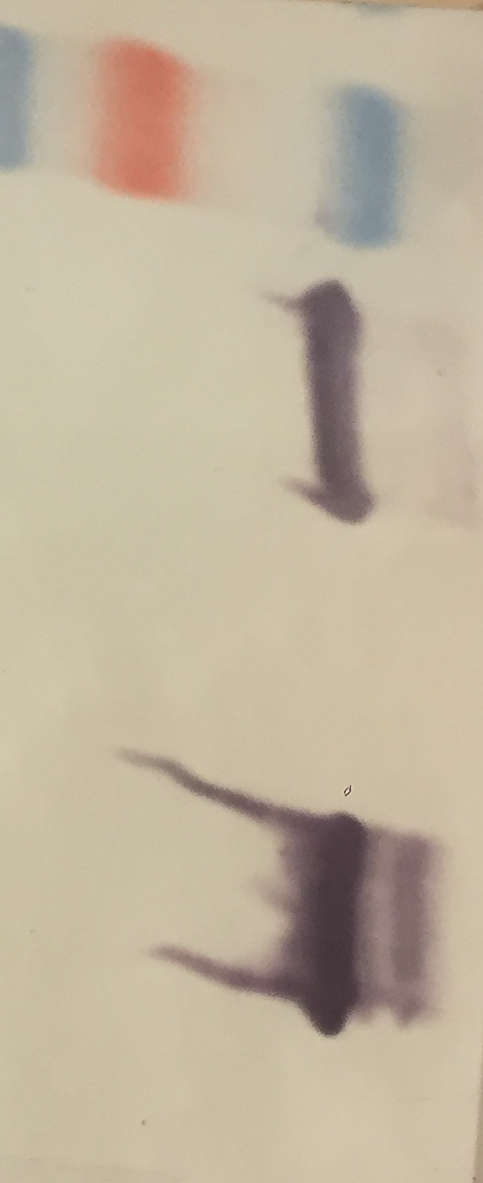


**GAPDH**

**Vimentin**

**HepG2 SNU449**

**35 kDa**

**55 kDa**

**Supplementary H4.** Membrane image for Vimentin protein expression in HepG2 and SNU449. Two bands were observed in SNU449.


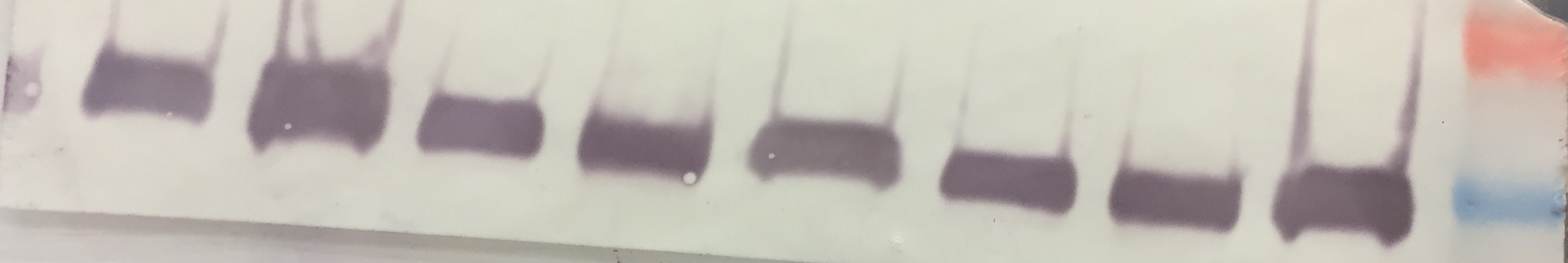


**HepG2 SNU449**

**B-tub**


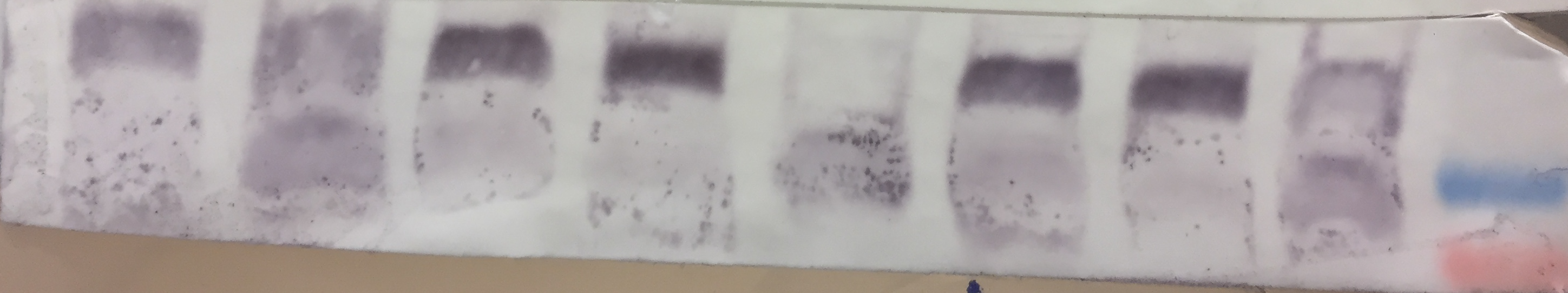


**SOX2**

**55 kDa**

**34 kDa**


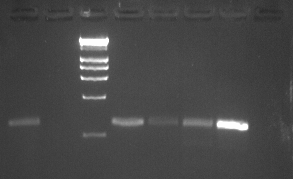

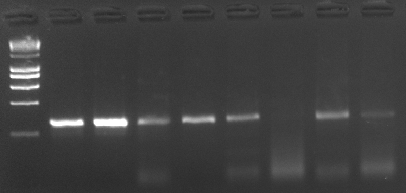


**SOX2**

**HepG2 SNU449**


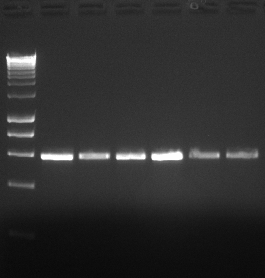


**GAPDH**

**598 bp**

**252 bp**

**A**

**B**

**Supplementary H5.** SOX2 mRNA and Protein Expression in HepG2 and SNU449 using RT-PCR and Western Blotting Respectively. GAPDH and β-tubulin were used as endogenous controls. A. RT-PCR analysis for SOX2 mRNA expression in HepG2 and SNU449. B. Membrane image for SOX2 protein expression in HepG2 and SNU449. Negatives were carried out for RT-PCR experiments.

**HepG2 SNU449**


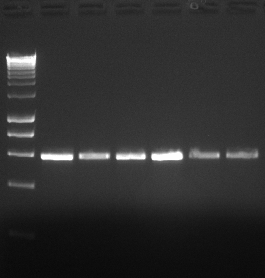


**GAPDH**


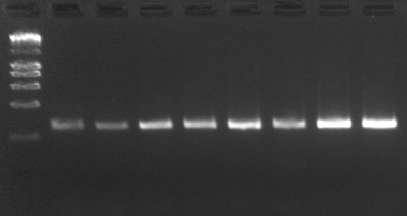


**FOXA2**


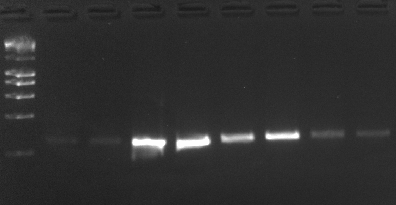


**VCAN**

**598 bp**

**270 bp**

**255 bp**

**Supplementary H6.** RT-PCR analysis for FOXA2 and VCAN mRNA expression in HepG2 and SNU449. Negatives were carried out for all experiments.
